# Ex-ante determinants of IPOs: A dataset for the Malaysian IPOs

**DOI:** 10.1016/j.dib.2025.111926

**Published:** 2025-07-25

**Authors:** Ali Albada, Soo-Wah Low, Rabie A. Ramadan, Khalid Al Qatiti, Muataz Salam Al-Daweri

**Affiliations:** a Faculty of Business, Sohar University, Sohar PC: 311, Oman; b Graduate School of Business, Universiti Kebangsaan Malaysia, Selangor PC: 43600, Malaysia; c Department of Information Systems, College of Economics, Management & Information Systems, University of Nizwa, Nizwa PC: 616, Oman; d Computer Engineering Department, Faculty of Engineering, Cairo University, Giza, Egypt

**Keywords:** IPOs, Signaling, Ex-ante, Information asymmetry, Malaysia, Fixed pricing method, Underpricing

## Abstract

This article introduces a comprehensive dataset for investigating the ex-ante determinants of Initial Public Offerings (IPOs) first-day initial returns, investor demand, and the information gap experienced by investors for listed firms on Bursa Malaysia over the period 2004–2021. The final sample comprises 350 IPOs priced using the fixed-price method, which were hand-collected from multiple sources. The data in this dataset plays an important role in providing insightful decision-making tips for prospective investors operating in an environment characterized by high information asymmetry. Furthermore, the dataset opens avenues for future research, such as cross-country studies exploring the impact of country-specific factors on IPO initial return outcomes.

Specifications Table

**Table:** 

Subject	Social Sciences
Specific subject area	*Initial Public Offerings (IPOs).*
Type of data	Excel spreadsheet.
Data collection	Most of the primary data was manually compiled from the Malaysian IPO prospectuses. Furthermore, the opening price, closing price, highest and lowest prices during the first trading day and the trading volume were gathered from the share investment website and cross-referenced with information provided by the iSaham website. The EMAS Index price information was provided by Yahoo Finance. Finally, the over-subscription ratio (OSR) data was gathered from various media outlets, including Star Online, the i3investor website, and the one-million-dollar blog.
Data source location	Malaysian IPO prospectuses: https://www.bursamalaysia.com/listing/listing_resources/ipo/ipo_summary Share investor website: https://www.shareinvestor.com/my iSaham website: https://www.isaham.my/ Yahoo finance: https://finance.yahoo.com/quote/FBMEMAS.FGI/ Star Online website: https://www.thestar.com.my/. i3investor website: https://i3investor.com/web/index. One-Million-Dollar blog: https://1-million-dollar-blog.com/
Data accessibility	Albada, Ali (2025) [1], “Bursa Malaysia IPO data 2004–2021″, Mendeley Data, V1, DOI: 10.17632/yn84h8jhxc.1. Full Link: https://data.mendeley.com/datasets/yn84h8jhxc/1
Related research article	Albada, A. Al-Daweri, M.S. Ramadan, R.A. Qatiti, K.A. Haoyang, L. & Shutong, P. 2024. [2]. Determinates of investor opinion gap around IPOs: A machine learning approach. Intelligent Systems with Applications, 23: pp. 200,420. Full Link: https://www.sciencedirect.com/science/article/pii/S2667305324000942

## 1 Value of the Data

• This dataset is unique due to its comprehensive hand-collected pre-IPO firm characteristics for 350 Malaysian firms listed on Bursa Malaysia over the period from 2004 through 2021.
• The meticulous compilation of these data provides a valuable resource for researchers exploring topics such as the fixed-price mechanism, environments characterized by high information asymmetry, signaling theory, and ex-ante determinants of IPO outcomes.
• The dataset not only facilitates a deeper understanding of these phenomena within the Malaysian context but also offers significant opportunities for future research. For instance, it enables cross-country analyses that explicitly examine the influence of country-specific factors on underpricing outcomes, thereby contributing to the broader literature on IPO pricing mechanisms and investor behavior in emerging markets.

## 2 Background

The data article complements the original research by providing a detailed and transparent account of the dataset used to investigate the signaling effects of ex-ante information in the Malaysian fixed-price IPO market. The dataset allows for reproducibility and enables researchers to validate, extend, or replicate the study. While the original article focuses on analyzing the ranking and impact of ex-ante signals on opinion divergence, the data article provides detailed description of the dataset’s variables, and collection methodologies in guiding future research on investor behaviors, IPO pricing efficiency and signaling value of ex-ante firms characteristics. Additionally, the dataset also allows researchers to apply other methodological approaches in exploring the dynamics of the Malaysian IPO market.

The dataset is valuable to researcher to address research gaps within the IPO domain, focusing on Malaysia, where the use of fixed-price method is prevalent. While most IPO research centers on developed markets like the U.S. and EU, this dataset expands research opportunities in the IPO underpricing literature of emerging economies through cross-country comparisons to capture a broader perspective in explaining the variations in IPO underpricing. The findings would be useful for investors to formulate investment strategies and for policymakers to improve IPOs regulatory framework. Additionally, the dataset highlights the role of underwriter reputation and auditor quality in IPO performance, suggesting the importance of improving due diligence and in the selection of underwriters and auditors. These contributions make the dataset a valuable resource for both academic and practitioner within the realm of IPOs.

## 3 Data Description

The dataset consists of 350 IPOs listed on Bursa Malaysia that utilized the fixed-price pricing mechanism. Table 1 provides a detailed overview of the variables included in the dataset, along with their definitions and classifications.

**Table 1:** Overview of dataset variables: definitions and types.

Symbol in the dataset	Variable	Definition	Type
Market_Condition	Market condition	A proxy for market confidence. High stock prices, market volume, and a high value of the stock market index reflects high market confidence. This indicates that initial returns will tend to be high during such a period.	Ratio
OSR	Over-subscription ratio	Captures immediate market reaction to the new issues. A higher ratio indicates higher investors' demand of the offered shares.	Ratio
Closing_Initial_Return	Closing First day initial return	IPO underpricing defined using price difference between offer price and closing price on first trading day.	Ratio
Primary_Initial_return	Primary First day initial return	IPO underpricing defined using price difference between offer price and opening price on the first trading day.	Ratio
Information_Gap	Investors’ information gap	Captures investors’ divergence of beliefs calculated as the price difference between the highest and lowest price on the first trading day.	Ratio
Listing_Board	Listing Board	During the study period the Malaysian IPO market consists of two listing boards, which are the Access, Certainty, Efficiency ("ACE") and Main Market.	Binary
Shariah_Status	Sharia Status	Issued shares that comply with Sharia-based screening, indicating that the listing firm operates in accordance with Islamic laws and rules, known as Sharia	Binary
Top_3_Investment_Banks	Underwriter reputation	The top three reputable underwriters identified based on the number of IPO issues undertaken, which are AmInvestment Bank, Alliance Investment Bank Berhad, RHB Investment Bank Berhad	Binary
Top_3_Auditors	Auditor reputation	The top three reputable auditors identified based on the number of IPO issues audited, which are Ernst & Young, KPMG, Crowe Horwath.	Binary
Retention_Ratio	Shareholder retention ratio	The percentage of shares retained by insiders after the IPO.	Ratio
Flipping_Activity	Flipping	The direct selling of an IPO allocation in the aftermarket during the first day of listing.	Ratio
Private_To_Total	Private Placements	Proportion of IPO issues allocated to institutional investors.	Ratio
Board_Size	Board size	The total number of individuals serving on the company’s board of directors.	Number
Board_Reputation	Independent Board members	The number of independent individuals serving on the company’s board of directors.	Number
Offer_Size	Offer size	The size of the IPO offering.	Natural log
Offer_Price	Offer price	The predetermined price at which shares are made available to the public.	Decimal

## 4 Experimental Design, Materials and Methods

The prospectus serves as a comprehensive source of pre-listing information about the issuing company and is a crucial document for understanding the IPO process. These documents are publicly accessible on the Bursa Malaysia website. The dataset spans from January 2004 to December 2021 and initially included 493 IPOs. To maintain focus on the research objectives, several exclusion criteria were applied. Specifically, only IPOs that utilized the fixed-price pricing method were considered; therefore, 39 IPOs that adopted the book-building method were excluded. The pricing mechanism employed for each IPO is explicitly stated in the respective prospectus documentation.

Subsequently, the study focuses exclusively on public issues, offer-for-sale transactions, or a combination of both. Other types of offerings—such as restricted or special issues—were excluded as they are not aligned with the research objectives and may yield less meaningful insights. Information regarding the type of offering is disclosed in the Malaysian IPO prospectus. In addition, Real Estate Investment Trusts (REITs) were excluded due to the distinct nature of their financial structures, which differ significantly from conventional IPOs and would not be suitable for comparative analysis. As a result of this exclusion, 104 IPOs were removed from consideration. Applying all the aforementioned criteria led to a final sample of 350 IPOs.

Moreover, EMAS Index price information was gathered from yahoo finance to help in calculating the market condition variables. Following Mahmood et al [3], the dataset measures market condition using the following Eq. (1):

(1) $$MKTCON_{Listing}=\left(\frac{PI_{LISTING}-PI_{OFFER}}{PI_{OFFER}}\right)$$

Where,

$PI_{LISTING}$ =EMAS Price Index on the day of listing, and

$PI_{OFFER}$ = EMAS Price Index on the day of offerings.

The OSR is based on the premise that IPO supply is often lower than investor demand. Consequently, when IPOs with higher OSR enter the market, they are more likely to experience strong upward pressure on their prices as investors rush to acquire limited shares. This excess demand typically translates into higher initial returns in the post-listing trading period. However, OSR data are not readily available in the prospectus or on the Bursa Malaysia website. To address this limitation, the authors sourced, validated, and cross-verified the OSR information using reports from reputable external sources, including Star Online, the i3investor website, and the One Million Dollar Blog.

Key variables extracted from the IPO prospectus documentation include listing board, offer price, offer size, board size, the number of independent board members, shareholder retention ratio, main auditing company name, principle underwriting company name, number of shares offered to institutional investors, and Sharia status. Listing board is measured as a binary variable that takes 1 to indicate shares listed on the ACE Market, and 0 otherwise. The Sharia status, a dummy variable assigned 1 if the shares hold Sharia-compliant status and 0 otherwise.

Furthermore, the names of the auditing and underwriting firms were used to assess the reputations of underwriters and auditors. The underwriter and auditor reputation were determined based on the number of IPOs each investment bank or auditing firm had underwritten or audited as the lead entity throughout the study period. This approach has been employed in prior studies, including those by Jelic et al [5] and Dimovski et al [4] for measuring underwriter reputation, and by Megginson and Weiss [6] for assessing auditor reputation. Additionally, the private placements ratio was calculated by dividing the number of shares allocated to institutional investors by the total number of shares issued during the IPO.

Additional information, including the opening price, closing price, highest and lowest prices during the first day of trading, and trading volume, was collected from the Share Investor website and cross-verified using data from the iSaham website. These data were utilized to compute various measures of initial return and market behavior. The primary measure of first-day initial return was calculated as the percentage difference between the opening price and the offer price, relative to the offer price. A secondary measure of initial return was also computed by dividing the difference between the closing price and the offer price by the offer price. The investor information gap was estimated by taking the difference between the highest and lowest secondary market prices on the first trading day, divided by the closing price. Lastly, the flipping activity variable was derived by dividing the trading volume on the first day by the total number of shares issued.

## Limitations

Not applicable

## Ethics Statement

The authors have read and followed the ethical requirements for publication in Data in Brief and confirm that the current work does not involve human subjects, animal experiments, or any data collected from social media platforms.

## CRediT authorship contribution statement

**Ali Albada:** Conceptualization, Writing – original draft, Data curation. **Soo-Wah Low:** Writing – review & editing. **Rabie A. Ramadan:** Methodology, Software, Formal analysis. **Khalid Al Qatiti:** Writing – review & editing. **Muataz Salam Al-Daweri:** Methodology, Software, Formal analysis.

## Data Availability

Mendeley DataBursa Malaysia IPO data 2004–2021 (Original data)
